# Variation in plastic responses to light results from selection in different competitive environments—A game theoretical approach using virtual plants

**DOI:** 10.1371/journal.pcbi.1007253

**Published:** 2019-08-21

**Authors:** Franca J. Bongers, Jacob C. Douma, Yoh Iwasa, Ronald Pierik, Jochem B. Evers, Niels P. R. Anten

**Affiliations:** 1 Centre for Crop Systems Analysis, Wageningen University, Wageningen, The Netherlands; 2 Plant Ecophysiology, Utrecht University, Utrecht, the Netherlands; 3 Laboratory of Entomology, Wageningen University, Wageningen, The Netherlands; 4 Theoretical Biology, Kyushu University, Fukuoka, Japan; University of Manchester, UNITED KINGDOM

## Abstract

Phenotypic plasticity is a vital strategy for plants to deal with changing conditions by inducing phenotypes favourable in different environments. Understanding how natural selection acts on variation in phenotypic plasticity in plants is therefore a central question in ecology, but is often ignored in modelling studies. Here we present a new modelling approach that allows for the analysis of selection for variation in phenotypic plasticity as a response strategy. We assess selection for shade avoidance strategies of *Arabidopsis thaliana* in response to future neighbour shading signalled through a decrease in red:far-red (R:FR) ratio. For this, we used a spatially explicit 3D virtual plant model that simulates individual Arabidopsis plants competing for light in different planting densities. Plant structure and growth were determined by the organ-specific interactions with the light environment created by the vegetation structure itself. Shade avoidance plastic responses were defined by a plastic response curve relating petiole elongation and lamina growth to R:FR perceived locally. Different plasticity strategies were represented by different shapes of the response curve that expressed different levels of R:FR sensitivity. Our analyses show that the shape of the selected shade avoidance strategy varies with planting density. At higher planting densities, more sensitive response curves are selected for than at lower densities. In addition, the balance between lamina and petiole responses influences the sensitivity of the response curves selected for. Combining computational virtual plant modelling with a game theoretical analysis represents a new step towards analysing how natural selection could have acted upon variation in shade avoidance as a response strategy, which can be linked to genetic variation and underlying physiological processes.

## Author summary

Plants are able to respond to changes in the environment. Particularly, plants can show different structural traits (e.g. stem height and leaf size) in different planting densities. These trait changes are the result of so-called plastic responses that can be induced by changes in the light spectrum. Although a great part of the physiological processes underlying these plastic responses have been identified, it remains unclear how these plastic responses, and variations therein, can be the result of selection. In this paper we analyse selection on different plastic responses within different dynamic competitive environments. We use a 3D virtual plant model that simulates realistic plant growth based on light absorption, photosynthesis and specific light signals that induce changes in leaf growth. The 3D model simulated various competitive vegetation stands consisting of plants with different plastic responses at different planting densities. We conclude that selection in different densities results in different plastic responses. We advocate that our modelling approach allows for analyses related to selection on plastic responses itself, instead of on specific trait values in different environments, and is therefore an important new step forward in understanding the role of plastic responses for plant performance.

## Introduction

In the course of evolution, plants have evolved traits often specific to a certain environment. When growth conditions change, the selection pressure on trait values change, which subsequently can change selection on genotypes. However, due to phenotypic plasticity one genotype can exhibit multiple phenotypes (i.e. multiple trait values) depending on environmental conditions [1–3], which helps a plant to survive across different environments. The extent to which plasticity is adaptive depends on the environmental conditions, the reliability of the cues that signal the (change in) environmental conditions, and the costs related to phenotypic changes and plasticity itself [4–7]. Although considerable genetic variation in plasticity has been documented in various species [8–11], it remains unclear how variation in plasticity is the result of direct evolutionary selection processes on a certain trait or a consequence of selection for other traits [12].

Evolutionary and ecological population models are widely used to explain genetic variation and species composition in different environments, and these models are often based on evolutionary game theoretical principles [13–17]. These models implicitly assume that variation in trait values is entirely due to genetic variation among genotypes. This implies that these models essentially predict selection for different genotypes in different environments when these environments select for different trait values. However, if plasticity would be considered in evolutionary game theoretical models as the ability of a genotype to change its trait value in response to environmental conditions, selection for different trait values in different environments would not necessarily lead to selection for different genotypes. In this paper we take the first step in exploring how variation in phenotypic plasticity could be the result of natural selection in different environments. Phenotypic plasticity is the result of plastic responses that are driven by physiological processes, and these responses are directly the result of environmental cues. To this end, the physiological processes underlying the plastic responses to environmental cues have to be quantitatively linked to trait values and to the performance of individual plants in various environments. Considering plasticity as a trait in itself and considering variation in plasticity across genotypes is required to analyse to which extent natural selection may have acted on variation in plastic responses.

In this study we focus on plastic responses to light competition in vegetation stands of varying planting density and associated neighbour-plant proximity. Plants growing at high density (close proximity to neighbour plants) typically exhibit greater elongation rates of leaf-supporting structures (i.e. internodes and/or petioles), reduced branching and greater leaf inclination angle than plants growing at low density [18–22]. One of the primary signals that induces these shade avoidance responses [23] is a reduction of the red to far-red ratio of light (R:FR), as plants selectively absorb red and reflect far-red light [20]. The reduction in R:FR light perceived by an individual plant is therefore considered a cue for neighbour proximity [24], reviewed in [23]; a low R:FR ratio signals that neighbours are close (high density) and a high R:FR ratio indicates that neighbours are farther away (low density). In addition, R:FR light conditions are also affected by the 3D structure of the canopy [22,25,26] and by self-shading [27]. These alternative causes of changes in the R:FR ratio may decrease the reliability of R:FR as cue for neighbour proximity and future light availability.

**Fig 1:**
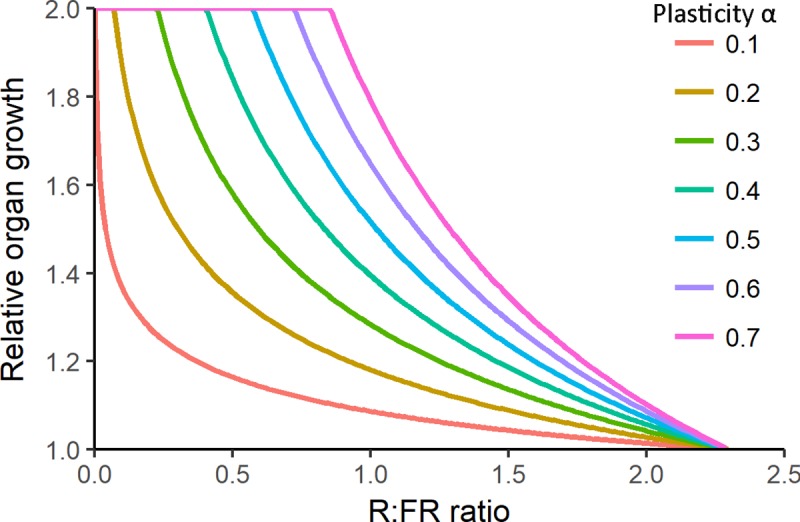
Visualization of different plasticity strategies. Plasticity strategies are presented with different values of α, and illustrate different shapes of the curve describing the relative organ response at various R:FR values (see Eq 1 in Methods). Different plasticity strategies tested within this study are created by increasing the value α from 0 to 0.7 with steps of 0.1 (see legend). Petiole and lamina responses are simulated by multiplying the organ (or organ growth) with this relative response value (see Methods for details). Plasticity strategy with α = 0 is a horizontal line at 1 (not visible in the graph) and indicates no plastic response to R:FR because a relative value of 1 does not change the organ size of growth rate. As the plasticity strategy α increases, strategies are referred to as being more sensitive to R:FR decrease.

To be able to analyse to which extent natural selection may have acted on variation in shade avoidance responses to R:FR, it is required to consider the feedback between the R:FR cue and the plant phenotype. Changes in R:FR induce responses at the organ-level that cause changes in plant architectural phenotype, which in turn affects light capture for growth. The changed phenotype, in turn, changes the light environment and associated R:FR conditions, inducing new sets of responses and this continues throughout plant development. This feedback can be captured in so-called functional-structural plant models ([28], also called virtual plant models) that can mechanistically simulate the interaction between plant 3D structure, growth, and the light distribution within the canopy [29,30]. While taking into account the phenotype-environment feedbacks created by the vegetation itself, R:FR induced organ-level plastic responses and variation within these responses can be realistically scaled up to whole-plant performance at vegetation level [31,32]. We utilize a recently developed and validated virtual plant model [27,31] that simulates Arabidopsis (*Arabidopsis thaliana*) rosette growth based on light absorption for photosynthesis and growth and induces phenotypic changes via a plastic response curve allowing plants to dynamically change their phenotype during growth. The current model simulates the consequences of specifically petiole and lamina plasticity [33] for whole-plant performance in different environmental conditions based on an organ level plastic response curve that describes the sensitivity of the relative petiole and lamina response to R:FR. The response curve is treated as a trait itself, and different shapes of the curve represent different plasticity strategies with the value *α* (Fig 1). Petiole plasticity entails petiole elongation in response to decreasing R:FR and can put the leaves in a higher strata of the canopy to increase light capture. Lamina plasticity entails lamina growth reduction in response to decreasing R:FR and can negatively affect light capture because it reduces the total lamina area. Therefore these two organ-specific plasticities are considered antagonistic.

**Fig 2:**
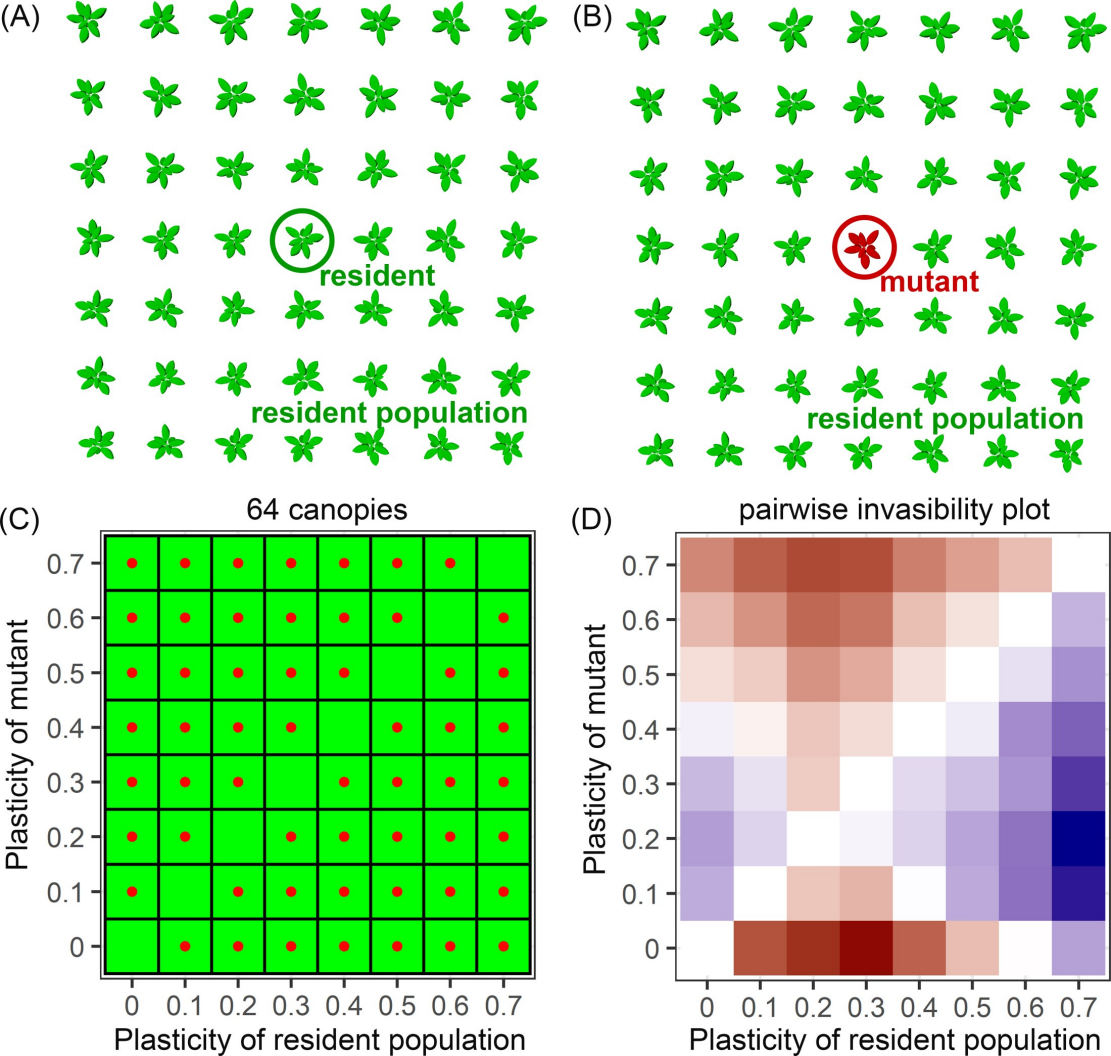
Representation of the modelling approach used in this study. Virtual plant canopies are simulated using a virtual plant model (previously described in ref. [27] and [31]). From every canopy, the simulated total biomass (proxy for performance) of the middle plant of the canopy is used for analysis to simulate the invasion of a rare individual within a big population and to avoid edge effects. If the mutant has the same strategy as the surrounding population (the ‘resident’ population), the performance of this mutant represents the performance of the resident population (A). If the mutant has a different strategy as the resident population, the performance represents the performance of the mutant within the resident population (B). Within this study, we tested a range of plasticity strategy values α from 0 to 0.7 (0, 0.1, 0.2, 0.3, 0.4, 0.5, 0.6 and 0.7), that represented different shapes of the relative organ response to the R:FR signal (Fig 1). A full matrix of combinations regarding the plasticity values for mutant within the resident population is simulated, resulting in 64 different canopies (C); eight canopies in which the mutant and resident population have the same plasticity strategy (green cells) and 56 canopies in which the mutant has a different plasticity strategy than the resident population (green cells with red dot). The 64 canopies representing this matrix are simulated for each planting density and for three scenarios (see Methods). The performance of each mutant-resident combination is used to construct pairwise invasibility plots, based on the invasion exponent of the mutant in each cell (D). The invasion exponent of the mutants is calculated by log (mean(mutant performance)/mean(resident performance)).

We combine this virtual plant modelling approach with evolutionary game theory (Box 1 and Fig 2) to analyse the extent to which variation in plastic responses could be the result of natural selection in different competitive environments. Different planting densities represent different competitive environments. Specifically, we search for convergence stable evolutionary stable strategies (cESS, [17,34]) at five different planting densities. A cESS (with strategy value α*) is a strategy that is evolutionary and convergence stable, which means that i) a resident population with trait α* cannot be invaded by a rare mutant with a trait value of α (both locally and globally) and ii) a mutant that has a trait value closer to α* than the trait value of the resident, can invade the resident population, if the resident population has any other value than α* [34,35]. In theory, the cESS definition also often requires the resident population to reach carrying capacity before invasion of a rare strategy [36,37]. However, in this study we assume that the resident population is at carrying capacity in the density tested as planting density is the environmental factor of interest.

**Box 1. How to analyse selection on variation in trait plasticity**

*Trait plasticity is the ability of a plant to change the absolute trait value in response to a change in the environment*. *Analysing selection on trait plasticity itself*, *i*.*e*. *the ability for the trait value to change gradually during development*, *is challenging*, *especially when the trait value and the environment are connected through a dynamic feedback loop*: *environmental conditions determine the trait value*, *which in turn affects environmental conditions*. *To analyse selection on trait plasticity related to dynamic light competition*, *we use virtual plant modelling and a plastic response curve (previously described in ref*. [31]*). The plastic response curve describes relative trait value change (e.g. relative petiole and lamina growth per day) related to an environmental variable (R:FR ratio) and can have different shapes, which we call different plasticity strategies (represented by α, see Fig 1*). *The shape of the response curve in combination with plant development and environmental conditions (e*.*g*. *light quality and quantity) determine the actual phenotype of the plant within the environment*. *Variation in the shapes of the plastic response curve can be linked to variation in physiological processes that underlie the response* [31]. *The shape of the curve can be subjected to selection processes because it is considered a single trait that can have different values for α*. *This way*, *the degree of a trait to respond to the changing environment is under selection*, *and not the absolute trait values that are expressed at specific moments in time within different environments themselves*.

**Presented modelling approach in a nutshell:**

1. *Different plant types are created with different shapes of the plastic response curve*, *also called plasticity strategies with value* α *(Fig 1).*
2. *Based on game theoretical principles*, *the virtual plant model simulates plants with different plasticity strategies competing for light in different competitive settings which results in plant performance per plasticity strategy per competitive setting (S3 Fig)*
3. *The simulated plant performance per plasticity strategy within a specific competitive setting is used to create pairwise invasibility plots from which a possible convergence stable evolutionary stable strategy (cESS) can be inferred (Figs 5 and 6)*.
4. *The cESS selected has a specific shape for the plastic response curve that represents a potential physiological process that enables a trait to gradually change value upon the perception of a changing environmental signal*.

To summarise, we ask the question to what extent natural selection may have acted on, or resulted in, variation in plastic responses by selecting different plastic shade avoidance response curves at different planting densities. We hypothesize that different plastic response curves will be selected at different planting densities if a given R:FR ratio signals a different level of future neighbour shading across these planting densities. In addition, we also explore the extent to which selection for plastic response curves depends on the cost trade-off associated with the petiole versus lamina responses to R:FR. The latter is expected to influence the selected plastic response curves because petiole and lamina responses have opposite effects on light capture and therefore influences plant performance.

## Results

### Plant growth in monomorphic populations without petiole or lamina plastic responses

**Fig 3:**
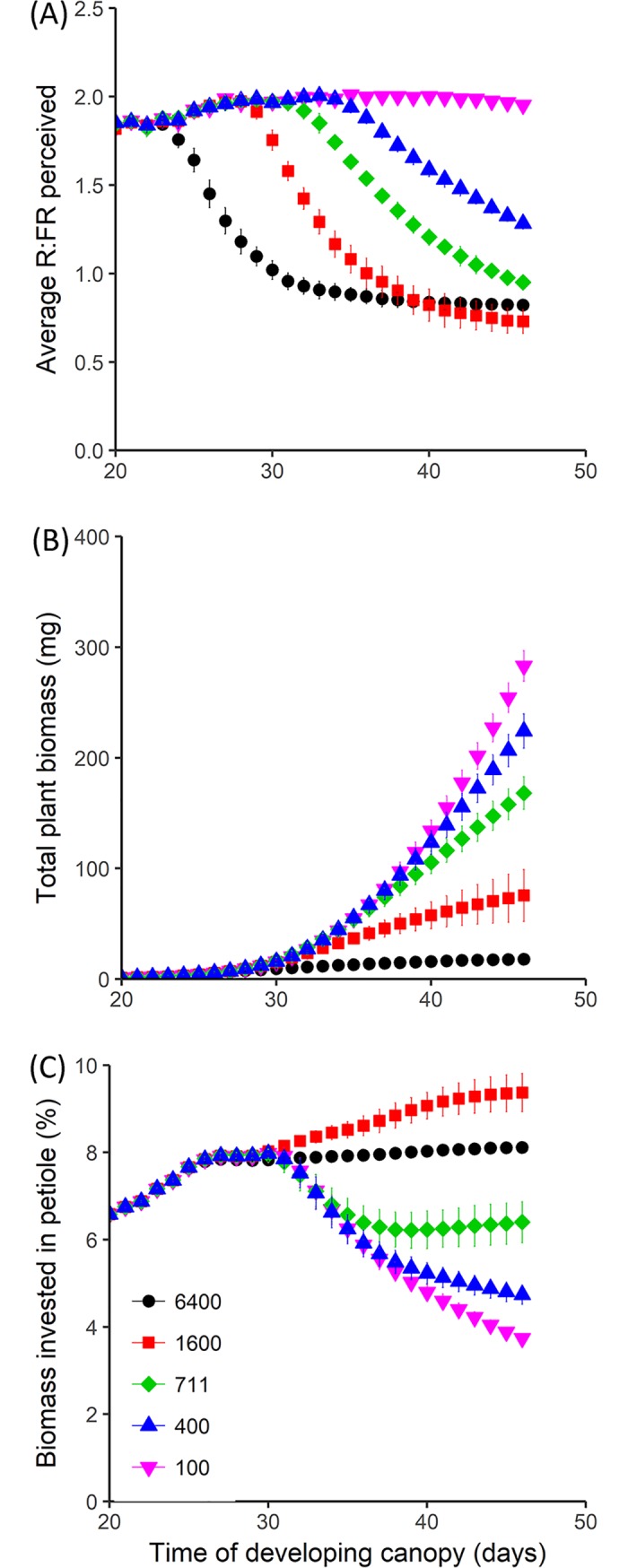
Simulated plant characteristics during canopy development of plants without petiole or lamina plasticity growing in monomorphic vegetation stands at five planting densities. Plants without petiole or lamina plasticity have the plasticity strategy α = 0. (A) Mean R:FR ratio perceived by the whole plant, (B) total accumulated biomass and (C) percentage of biomass invested in the petioles. Plants grew from seed for 46 days at five different planting densities; 100, 400, 711, 1600 and 6400 plants m^-2^, see legend. Average perceived R:FR ratio was calculated by dividing the total amount of perceived red light with the total amount of perceived far-red light. Percentage biomass invested in petioles was calculated based on whole-plant biomass which entails petioles, laminas and the single root. Data represent mean ± SD (n = 20).

To illustrate how plant growth in the virtual Arabidopsis model depends on planting density (Fig 3), we simulated the growth of plants within monomorphic vegetation stands consisting of plants that do not exhibit R:FR induced plasticity related to petiole elongation or lamina growth reduction (plasticity strategy *α* = 0 in Eq 1) at different densities. During canopy development, interaction between leaves of neighbour plants (see Methods and ref. [31]) resulted in increased leaf angles and lamina growth resulted in increased total leaf area index (Supporting Information S1 Fig). Together these processes decreased the average R:FR ratio perceived by the plants (Fig 3A). In addition to the decreasing R:FR ratio, light availability per individual plant also decreased during canopy development due to the presence of neighbour plants. Therefore, individual plant growth, represented by total accumulated biomass, was, at the end of canopy development, lower at high than at low densities (Fig 3B). Biomass allocation to different organs varied over time and varied with density because allocation of carbon to growing organs depended on the total available carbon due to light capture, and on the relative growth rates and the total number of growing organs at any time step (for model description see Methods). Under weak competition for light (low densities), the percentage of biomass allocated to the petioles decreased near the end of canopy development (Fig 3C) because sufficient carbon was available to reach potential growth of petioles and laminas and leftover carbon was additionally stored in laminas and the root. On the other hand, under strong competition for light (e.g. at high densities of 1600 and 6400 plants m^-2^) there was relative low carbon available for growth of all organs. From this low available carbon, a higher fraction was invested in petioles and relative little in laminas and the root, by which the percentage of carbon allocated to the petiole increased (although slightly at 6400 plants m^-2^).

### The role of plastic responses in biomass allocation patterns and total plant biomass

**Fig 4:**
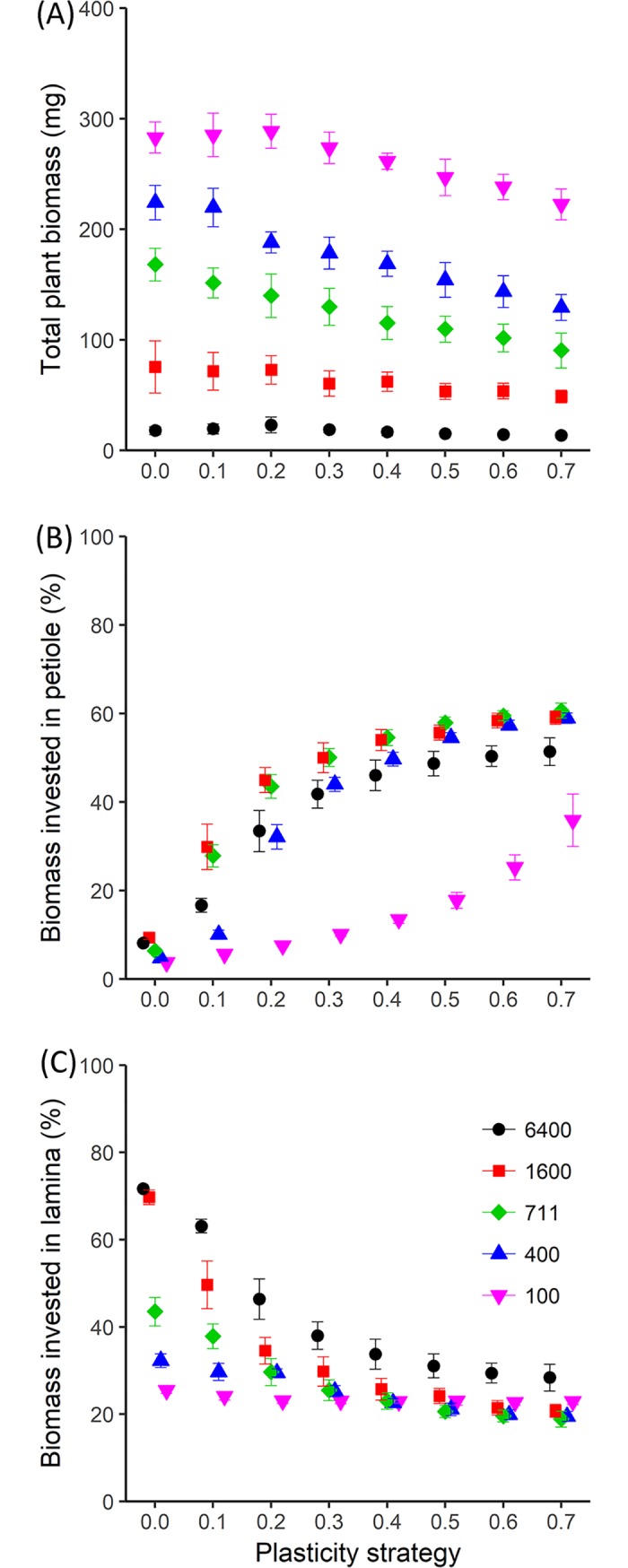
Simulated total biomass and biomass allocation to petioles and laminas for plants with different plasticity strategies growing in monomorphic vegetation stands at five planting densities. Plasticity strategy refers to the shape of the plastic response curve that describes the petiole and lamina response to R:FR (Fig 1). (A) Total plant biomass, (B) and (C) percentage biomass invested in petioles and laminas at the end of canopy development, depending on plasticity strategy (x-axis) and planting density (colours and symbols, see legend). Data represent the mean ± SD (n = 20).

To illustrate how the plasticity strategy (shape of the plastic response curve) affects the total plant biomass and the organ specific biomass allocation, monomorphic vegetation stands with different plasticity strategies were simulated (according to the Average scenario). Total plant biomass decreased, in general, with increasing plasticity strategy (Fig 4A), generally being highest for non-plastic individuals. This suggest that monomorphic populations (all individuals having the same plasticity) with low or no levels of plasticity generally perform better than populations with high levels of plasticity. There were exceptions to this trend, especially being that at the highest density (6400 plants m-2) biomass was higher at 0.2 plasticity level than for the 0 level (non-plastic plants). This result suggests that at very high densities some level of plasticity may have a benefit for population level light capture and performance.

During canopy development the plasticity strategy influenced the biomass allocation to petioles and laminas, which resulted in finally different percentages of biomass invested in petioles and laminas (Fig 4B & 4C). Plants with a high plasticity strategy (high α value) allocated relatively more biomass to the petioles and less to the laminas because these plants induced petiole and lamina plasticity at a relatively high R:FR earlier during canopy development. The differences between plasticity strategies on organ biomass allocation are represented in the organ-rank specific sizes, which embody the rosette phenotype of the plant (S2 Fig). The final percentage of biomass allocated to the petioles or laminas was different between planting densities because the dynamically changing R:FR influenced the plastic responses on top of the differences in light availability during growth and the organ specific growth rates, as illustrated previously (Fig 3).

### Selection for cESS at different densities (Average lamina responses scenario)

**Fig 5:**
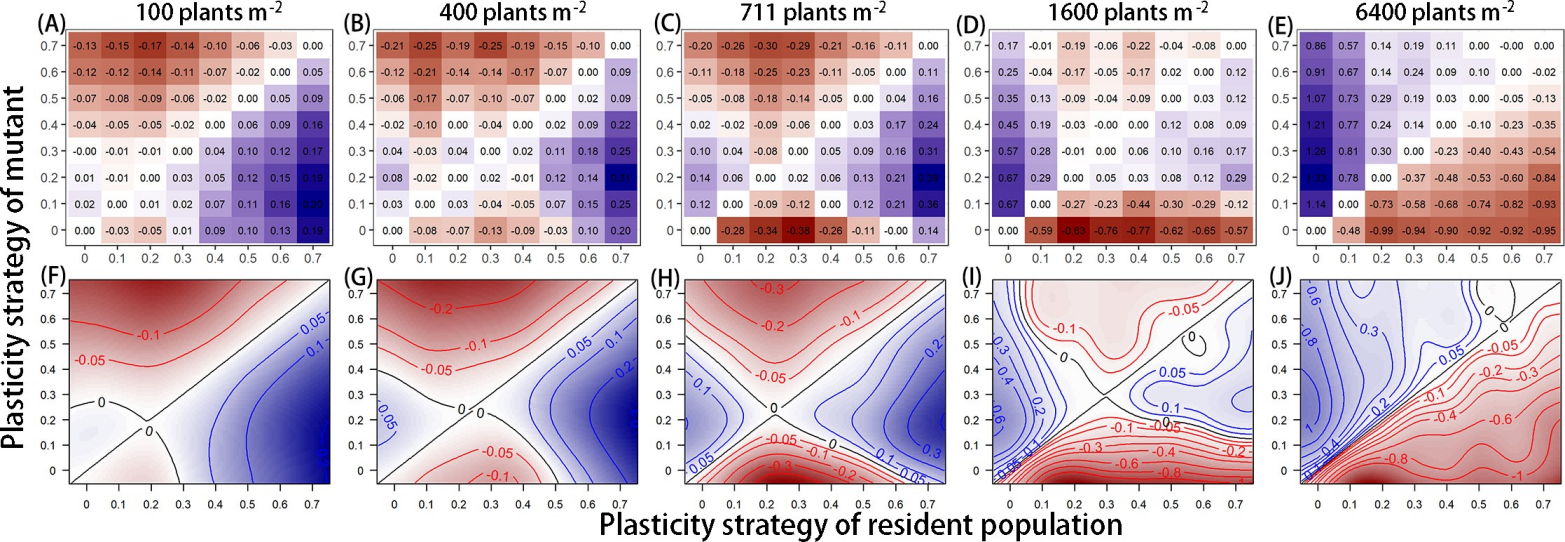
Discrete and smoothed pairwise invasibility plots at five planting densities, while lamina responses were average (Average scenario). The Average scenario consists of average lamina responses compared to petiole responses, which is represented by *n* = 2 in Eq 2, see Methods. Values within the plots represent the invasion exponents of the mutant within the corresponding resident population with a corresponding colour-gradient going from dark red (negative) to dark blue (positive). (A-E) Discrete pairwise invasibility plots present the simulated invasion exponents of the mutant for the eight values of plasticity strategy tested. See S4 Fig for the corresponding confidence intervals. (F-J) Smooted pairwise invasibility plots are based on the interpolation of the simulated performance values using a non-linear smoother (See Methods for details). See the main text for detailed explanation of how to read pairwise invasibility plots. In short, the point where the identity line and the second isoclines (if present) intersect corresponds to a singular strategy that, in this case, represents a cESS.

**Fig 6:**
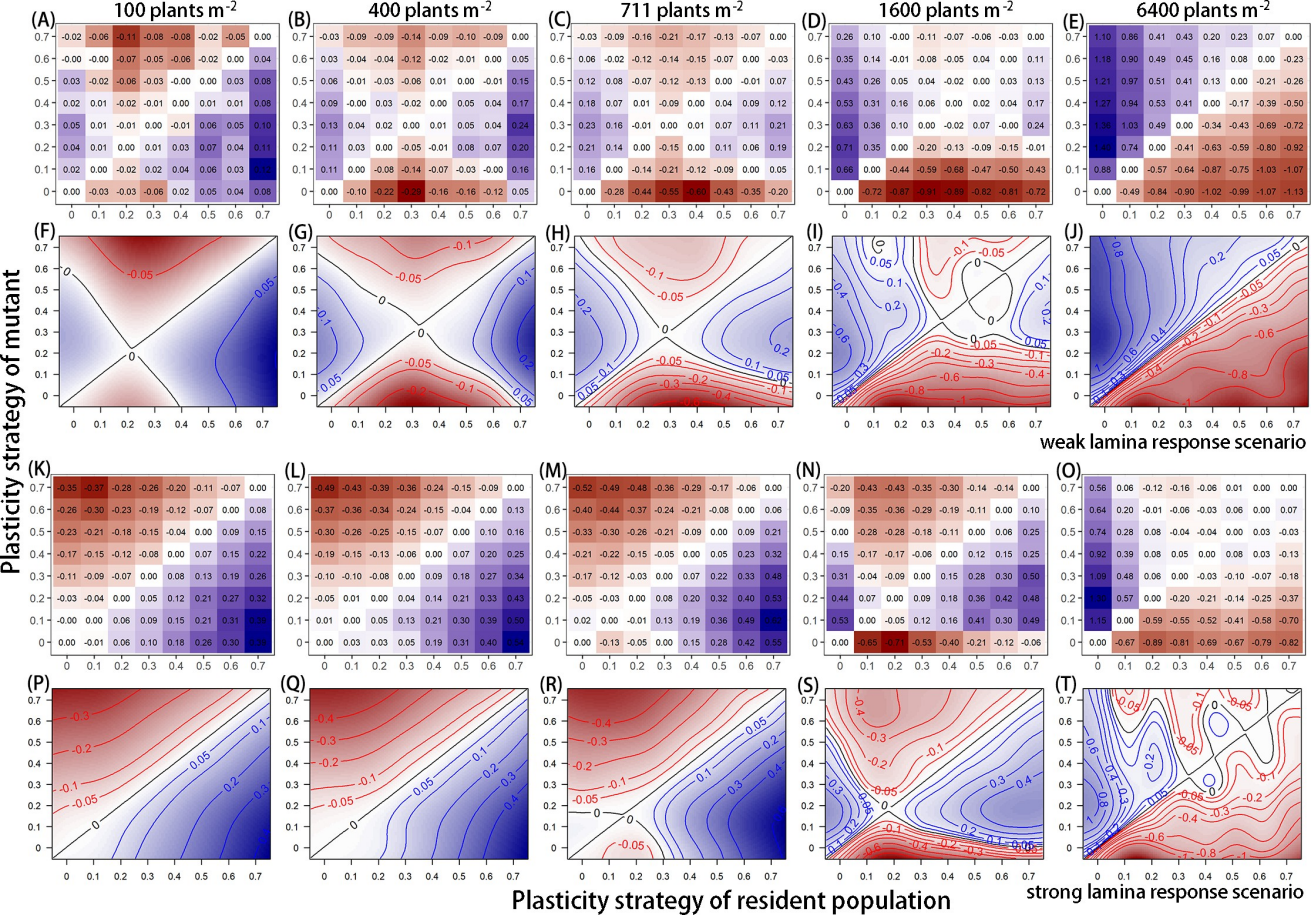
Discrete and smoothed pairwise invasibility plots at five planting densities for the Weak and Strong lamina response scenarios. In the Weak and Strong scenarios, the strength of lamina responses was decreased or increased, respectively, relative to petiole responses while the plants still had the same plasticity strategy value. In the model settings these scenarios were created by changing the *n* value in Eq 2, see Methods; *n* equals 0 or 4 for the Weak and Strong scenario respectively. Values within the plots represent the invasion exponents of the mutant within the corresponding resident population with a corresponding colour-gradient going from dark red (negative) to dark blue (positive). (A-E and K-O) Discrete pairwise invasibility plots present the simulated invasion exponent of the mutant for the eight values of plasticity strategy tested. (F-J and P-T) Smooted pairwise invasibility plots are based on the interpolation of the simulated performance values using a non-linear smoother (See Methods for details). See the main text for detailed explanation of how to read pairwise invasibility plots. In short, the point where the identity line and a second isoclines (if present) intersect corresponds to a singular strategy that could represent a cESS.

To determine how natural selection may have acted on variation in the plastic response curve, and how this selection may depend on the competitive environment, we performed an evolutionary game theoretical analysis in which we searched for cESS (see introduction for the definition) at five planting densities. The virtual plant model simulated the performance of a mutant within a resident population for different combinations of plasticity strategies for the mutant and the resident population (see Fig 2C) at five planting densities (S3 Fig). These values were used to calculate the invasion exponents of the mutants, which were used to construct pairwise invasibility plots: positive and negative values of the invasion exponent relate to positive (blue) and negative (red) invasibility (Figs 5A–5E, 6A–6E and 6K–6O). To aid the interpretation of these pairwise invasibility plots and help identifying possible cESS, the values of the performance of the mutant within a resident population were interpolated with a non-linear smoother after which the invasion exponent of the mutant was calculated (Figs 5F–5J, 6F–6J and 6P–6T, see Methods for details).

By definition, the identity line in the invasibility plots represents the case where the performance and strategy value of the mutant are identical to the performance and strategy value of the residents (1:1 line). A second isocline (if present) represents the plasticity values where the performance of the mutant equals that of the resident but without the mutant and resident having the same plasticity value. The point where the identity line and the isocline intersect corresponds to a singular strategy that could represent a cESS. In graphical terms the singular strategy is a cESS (with value a*) when moving up or down from the identity line no mutant has higher performance compared to the resident performance (positive invasion exponent of the mutant); and when starting with a resident population that is left of a*, a mutant closer to a* should have a higher performance than the resident population (the same holds for residents right from a*). The extent to which the mutant can be closer to a* is determined by the second isocline. If the region between the isocline and the identity line does not include the horizontal line through a*, a singular strategy cannot invade all resident populations directly, but only through a series of stepwise mutations. Alternatively, the singular strategy could represent a branching point or evolutionary repeller (see [34] for an accessible treatment).

The pairwise invasibility plots based on the average petiole and lamina responses (Fig 5, see S4 Fig for the confidence intervals) show possible cESS at all densities, except for 6400 m^-2^. The cESS per density corresponds with different plasticity strategy values; higher plasticity values were selected at higher planting densities (Fig 5). This represents selection for plastic response curves with increased sensitivity for R:FR at higher densities. At the highest density (Fig 5J) a second isoclines is not present, by which a possible cESS could not be determined within our tested values. However, both the discrete and smoothed invasibility plots suggest that selection would result in plants with a high plasticity strategy (high R:FR sensitivity) probably beyond the tested values. At 1600 plants m^-2^ (Fig 5I) the pairwise invasibility plot has a complex shape around plasticity values of 0.5 and 0.6. The calculated values with their confidence intervals (Fig 5D and S4D Fig) show that the invasion exponents of plants with plasticity 0.5 and 0.6 in resident populations of 0.6 and 0.5 respectively are around zero, which means that more simulations are required to determine the precise value of the invasion exponents at that part of the pairwise invasibility plot.

### cESS affected by the balance between petiole and lamina responses

Finally, we analysed the extent to which the above-mentioned cESS per density depended on the relation between petiole and lamina responses. In two additional scenarios, we either decreased (Weak scenario) or increased (Strong scenario) the lamina responses relative to petiole responses upon perception of R:FR (parameter *n* in Eq 2, see Methods). The balance between petiole and lamina responses, although both have the same plasticity strategy, relates to a trade-off regarding light capture during competition; increased light capture due to longer petioles versus decreased light capture due to smaller lamina area. Although not all pairwise invasibility plots identified a single cESS, the balance between petiole and lamina responses did clearly affect the mutant’s invasion exponents at different densities (Figs 5 and 6). When lamina responses were relatively small, there was selection towards plants with a slightly higher plasticity strategy value, evidently at the three lowest densities (compare Weak scenario Fig 6A–6J with Average scenario Fig 5). Higher plasticity strategy values relate to more sensitive plastic response curves. At 1600 plants m^-2^ (Fig 6I) there were three intersections between isoclines and the identity line, which suggests that the invasibility environment has a complex shape. However, similar as for the Average scenario, the confidence intervals of the invasibility exponents of plants with plasticity strategy value of 0.3 up to 0.6 included zero (S4I Fig), which suggest that strong conclusions about the invasibility of these mutants within their corresponding resident populations cannot be made with confidence based on the data. Invasibility exponent values around zero suggest that not one single plasticity strategy with a narrow range value would eventually dominate the population, but that plants with a broader range of plasticity values could persist together. At 6400 plant m^-2^ (Fig 6J) there was no second isoclines, which means that within the tested values no conclusive cESS could be determined. However, the invasibilty plot suggests that a potential cESS lies higher than the 0.7 tested here.

With relatively strong lamina responses there was selection for plants with a lower plasticity strategy compared to weak and average lamina responses (Figs 5 and 6). Although at 100 and 400 plants m^-2^ (Fig 6P and 6Q) there was no conclusive cESS, we expect that plants with a zero or negative plasticity strategy would be selected for. Negative plasticity strategy values would indicate that plants would invest less carbon in petioles and more carbon in laminas in response to lower R:FR. At 6400 plants m^-2^, the second isocline crossed the identity line multiple times (Fig 6T). This suggests again that the invasibility environment could be complex, and that not one single plasticity strategy would dominate a population at 6400 plants m^-2^. More simulations of various mutant-resident combinations within our tested range could verify the exact shape of the complex invasibility environment. Additional replication simulations could also verify that the observed complex invasion environments are not due to stochasticity in the model outcomes.

## Discussion

In this study, we analysed if variation in shade avoidance plasticity could be the result of natural selection in different environments. We did this by testing if different planting densities selected for different plasticity strategies that embodied petiole and lamina responses to a decrease in the ratio between red and far-red light (R:FR). Using a virtual plant model allowed us to scale from organ-level plastic responses and variation therein, to whole-plant phenotype and performance at population level. Organ-level plasticity was induced by changes in the locally perceived R:FR ratio by using a plastic response curve, and allowed plants to dynamically change their phenotype during the growing season, depending on the distance, size and plasticity strategy of neighbour plants. The study shows that different planting densities can select for different response curves, as reflected in the different convergence stable evolutionary stable strategies (cESS); with increasing density more R:FR sensitive plastic responses are selected for. This is in spite of the fact that plants use R:FR variation as a signal of plant density itself. Our findings suggest that variation in shade avoidance responses and variation in signal-sensitivity observed in plants could, in some cases, be the result of selection processes at different planting densities. Since density typically varies over growing seasons and in space and different densities select for different response curves, this could have acted to maintain genetic variation in plastic responses, and could help explain the large variation in plastic responses that is observed within species. In addition, the study shows that the balance between lamina and petiole responses affects the selected plastic responses curves because petiole and lamina responses had antagonistic influences on light capture. Plants with lower signal sensitivity were selected for when inducing plastic responses had relatively high negative consequences for light capture.

### Higher R:FR sensitivity selected at higher densities

We hypothesized that different plasticity strategies would be selected at different planting densities if a given R:FR value signals different levels of future neighbour shading across planting densities. Our results show that selection would lead to different response curves at different planting density (Fig 5). We argue that this may be partly due to the fact that the severity of future neighbour shading (expressed in reduced photosynthetic active radiation) that is signalled by a given R:FR value, differs between planting densities. The amount of photosynthetic active radiation received by laminas whose petioles perceived a R:FR around 1.0 decreased with density (S5 Fig). In low density stands, a drop in R:FR during canopy development is mainly caused by self-shading, whereas in high density stands, R:FR reduction is more strongly determined by neighbour-shading [27]. This means that at different plant densities, a given R:FR ratio has different meanings regarding light availability and the level of impending light competition. R:FR is thus not a fully reliable cue for future shading over a range of densities. This prevents selection for one single plastic response curve over a range of densities. In low densities, when light competition is low and R:FR changes are mostly caused by the plant itself, a less sensitive response to a given R:FR is favourable to avoid relative long petioles and small laminas that have negative consequences for light capture and thus plant performance [19]. In high densities, a more sensitive response curve is favourable to create long petioles that can avoid neighbour shading and increase light interception for plant growth and performance.

Our conclusion, that selection at different densities can result in variation in phenotypic plasticity if the signal is unreliable for future environmental conditions, agrees with previous studies that concluded that signals can have different meanings in different environments, such as R:FR values in open versus closed-canopy forests [10,38,39], which could explain the observed variation in plasticity. Our model approach is different from these studies in that it focussed on a plastic response curve that represented the potential to induce relative trait changes instead of absolute trait values and value differences upon an environmental change. Our plastic response curve could be linked more readily to underlying genetics and physiological processes, as shown for Arabidopsis mutants deficient in transcriptional regulators or hormones [31]. Consequently, our modelling approach represents a step forward in linking selection processes to the genetic basis and physiological processes underlying phenotypic plastic responses.

### Balance between antagonistic responses affects plasticity selected for

We explored the extent to which selection for plastic response curves at different densities would depend on the trade-off between petiole and lamina responses that respectively would relate to increased and decreased light capture. Changing the strength of lamina responses relative to petiole responses influenced selection for the response curves over the full range of planting densities. Plants with higher plasticity strategy values were selected for when petiole responses were associated with lower lamina responses (Figs 5 and 6, Weak scenario compared to the other scenarios). This indicates that when inducing plastic responses has lower negative consequences due to lower lamina growth reduction, selection can result in higher R:FR sensitivity. In contrast, plants with lower plasticity strategies were selected for when petiole elongation was associated with a stronger reduction of lamina growth in response to R:FR (Figs 5 and 6, Strong scenario compared to other scenarios). Although at the lowest densities no cESS was found within the tested strategies, the pairwise invasibility plots suggest selection in the direction of plants with zero or negative responses to R:FR. A negative value would indicate that plants would invest less carbon in petiole growth and more in lamina growth in response to lower R:FR. However, based on experimental work [33] we conclude that these inverse petiole and lamina responses to decreasing R:FR are not plausible in Arabidopsis. In general, we conclude that plants without plastic responses to R:FR would be selected for (i.e. plants with low sensitivity for R:FR) when performance consequences of showing plasticity are too negative. This is in accordance with other theories. For example, studies related to the evolution of phenotypic plasticity [6,40] state that selection would favour non-plastic responses if costs of inducing plastic responses are high. In addition, error management theory [41] would predict that when phenotypic responses have high costs, selection would favour less sensitive response curves responsible for the plastic response. Altogether, our results are a quantitative example of the influence of cost trade-offs related to plastic responses on the selection for specific plastic responses.

### Modelling choices within the virtual plant model

The 3D virtual Arabidopsis model did not include other light signals than R:FR that can signal neighbour proximity and future shading [42]. For example the combination of low blue and low R:FR can indicate stronger shading and therefore can induce stronger shade avoidance responses than either light treatment alone [43]. Importantly, low blue and low R:FR signals can be created either by increased vegetation density of plants with roughly similar sizes or by tall trees in a closed-canopy forest. This suggests again that the reliability of an environmental cue to induce a response depends on the competitive environment. The location of signal detection on the plant can also affect reliability. For example, perception of low R:FR at the lamina tip is more reliable as cue for neighbour-proximity than R:FR perception at the petiole [27]. The regulation of multiple signals and their interactions are still poorly understood and need to be further studied to better understand selection for organ-specific plasticity under natural conditions.

In addition, besides petiole and lamina plasticity, our simulations did not consider responses to R:FR other than leaf angle increase (see Methods). Other responses such as specific leaf area increase [44], flowering time acceleration [8], root development reduction [45] and defence reduction [46] can also affect plant competition for light [32,47,48] and can thus influence selection for specific plastic response curves. We also did not consider any form of mechanical penalty on developing longer petioles, which would occur in natural systems and can affect plant performance in a density dependent manner. For example, the vulnerability to mechanical damage or hydraulic limitations for longer petioles can depend on density; in high density canopies, leaves can get mechanical support from surrounding leaves or protection against wind, by which plants have a lower risk of mechanical failure even if investment in supporting tissues is low. In low densities, this protection is low (or absent) and investment in longer petioles requires additional carbon allocation for petiole stability, which may affect final plant performance. If inducing phenotypic changes has higher fitness costs in low than in high planting density, we would expect an even larger effect of planting density on the selected response curves than what we found in the present study.

### Evolutionary and ecological stable strategies

Although we considered a wide range of planting densities (regularly spaced), we did not consider that inter-plant distances within a natural vegetation is normally heterogeneous in space, which would make the light environment even more variable. Our result that different densities select for different plasticity strategies, suggests that when density is more variable in time and space transient evolutionary dynamics may prevail allowing different strategies to persist transiently [49]. Performing an analysis in which the distance between neighbour plants within the vegetation is heterogeneous or the density between successive generations is variable, could be the subsequent step towards identifying if and how many genotypes could persist over time.

In theory, the definition of an cESS requires a population to reach carrying capacity before invasiveness of a rare mutant is tested [36,37]. But in our analyses planting density was the environmental factor in question, and could thus not be changed as part of the analyses. We thus implicitly assumed that the different densities reflected different carrying capacities as determined by the overall environment (e.g. by resource availability). A consequence of the fact that different planting densities resulted in different cESS could be the following: if a given plastic response strategy is a cESS at a specific density, but the carrying capacity is at another density, then one may expect some kind of transience. Eventually it could be expected that different plastic response strategies will persist transiently if density changes strongly between years, or it could be expected that one single strategy would persist if the density stays constant around the carrying capacity of the population. It would be challenging and interesting to consider evolutionary and ecological dynamics while exploring the evolution of plastic responses in future studies.

### Conclusion

Our analyses indicate that different planting densities can select for different plastic response curves that represent different R:FR sensitivity for petiole and lamina responses. This is consistent with the considerable genetic variation in shade avoidance responses observed in Arabidopsis and in several other species [8–11]. This result seems in part to be explained by the reliability of the R:FR signal as a cue for future neighbour-shading that varies per density. In addition, selection for specific shapes of the plastic response curve is influenced by the trade-off between responses that have generally positive (petiole elongation) versus negative (lamina growth reduction) consequences for light capture and therefore plant performance. Combining virtual plant modelling and evolutionary game theory is a new step toward analysing how phenotypic plasticity, and the underlying sensitivity to an environmental signal, can affect the composition of genotypes over a range of environments. Promising next steps could be including responses to multiple light signals, considering environmental dependence of inducing phenotypic plasticity and regarding environments to be more variable in time and space.

## Methods

For this study, a functional-structural plant model of Arabidopsis rosette growth and development was utilised [27,31], built in the simulation platform GroIMP v1.5 (https://sourceforge.net/projects/groimp/). These types of virtual plant models have been proven strongly capable of predicting photosynthetic active radiation and R:FR distribution and plant architecture in stands of different planting densities for various species [32,50,51], including Arabidopsis [22,27,31]. This Arabidopsis model can simulate competitive interactions between Arabidopsis genotypes that differ in sensitivity to R:FR, a key element of this study, and has been validated [31]. The essence of this model is that all individual plants within the canopy grow as a function of the light they absorb, but at the same time create the light environment itself. Therefore, simulating various plant types with different phenotypic responses will determine the specific light environment within the canopy, which in turn will have repercussions for plant growth.

### Basic model layout

Here we summarise the model description and specify the most important model components, assumptions and choices. More details can be found in ref [31]. Simulated Arabidopsis plants emerged from seeds and grew for 46 days into an adult rosette plant with multiple leaves (petiole and lamina) produced in a spiral pattern, and a single root. The leaves captured light for photosynthesis and were also sinks for carbon. The root only functioned as carbon sink and had no effect on aboveground growth. Organ initiation (e.g. time between leaf emergence) and geometric representation such as orientation of the leaves and the shape of the leaves were simulated using empirical relationships. Plant growth was driven by total carbon assimilation and organ-specific carbon allocation and plastic responses induced by the R:FR environment.

The simulated light source emitted photosynthetic active radiation, red and far-red light and in each model time-step (representing 24 hours) these stochastic emitted light rays were reflected, transmitted and absorbed by the petioles and laminas individually according to their wavelength-specific spectral properties. The light source emitted an R:FR ratio of 2.3, photosynthetic active radiation with an intensity of 220 μmol m^-2^ s^-1^, and total daily light intensity was calculated based on 9 hours light per day, representing growth chamber conditions under which validation experiments had been carried out [31]. Plants were placed in regular places grids with different inter-plant distances to create different planting densities: 100, 400, 711, 1600 and 6400 plants m^-2^, which bracketed the densities in the validation experiment [31]. Border effects were minimized by using the plot replication functionality of GroIMP; canopies were replicated 20 times in the *x* and *y* direction and light conditions were calculated and averaged for these 400 canopies.

The local light environment perceived by the individual plants (at the organ level) was created by the specific 3D structures of all plants within the canopy itself. Every individual organ absorbed light which enabled the model to calculate the light partitioning over all individual plants within the canopy. This way, we did not have to make assumptions about light partitioning over individuals with different trait values, as has been done in most other game theoretical light competition models [52–54]. Thus, our model simulates competition for light as an emergent property and not an input parameter. Total accumulated biomass stored in root, laminas and petioles after 46 days of growth was used as a measure of plant performance. The rationale is that seasonal biomass scales with seasonal seed production, which for an annual plant like Arabidopsis equates to life time reproduction [55]. Generally for light-demanding species like Arabidopsis, under light competition this correlation is very strong [21,56,57]. Plants could not die during canopy development, they only stopped growing when light capture was insufficient.

The laminas individually absorbed photosynthetic active radiation that was converted into growth substrates via photosynthesis, assuming a negative exponential light response curve [58]. Total growth substrates per individual plant were summed into a central pool and subsequently partitioned over all growing organs through the relative sink strength principle [59]. The relative sink strength of an organ was expressed as a fraction of total plant sink strength, and determined the demand for substrates for each organ in relation to its age. Organ sink strength was defined as its potential growth rate and calculated using the beta growth function [60]. The beta growth function calculated the growth rate at organ age based on measured maximum organ size and duration of organ growth. Leaves senesced after 40 days of age. Thus primarily, the organs individually grew in time and 3D space based on the allocated substrates they received based on their relative sink strength. This means that when petioles receive more substrates due to an increase in relative sink strength, automatically other sinks such as leaves and roots will receive less.

### Lamina and petiole plasticity

Lamina and petiole growth was influenced by shade avoidance responses that were induced by changes in the R:FR ratio. In this study we focus on the petiole elongation and downregulation of lamina growth [33]. Leaves also showed leaf angle responses due to neighbour proximity and R:FR conditions, see ref [27,31], but this was not changed in between simulations. Leaves increased their angle with 16 degrees per time-step when the distance between neighbouring leaves was smaller than 2 mm (mimicking touching of leaves [22]) or when R:FR perception at the lamina was below a threshold of 0.5. These settings were chosen based on wild-type Arabidopsis responses and allowed plants to induce leaf angle increase depending on planting density. Petiole elongation and lamina growth downregulation responses occurred every time-step based on the response curve that illustrates the relationship between relative organ growth and R:FR perception at the organ itself (Fig 1). The response curve was found by fitting an empirical relationship through experimentally obtained petiole elongation data [31]:

$$F=min\left(2,\;{\left(\frac{R:FR}{RFRcontrol}\right)}^{-\alpha }\right)$$ (1)

*F* is the relative organ growth factor. R:FR and RFRcontrol represent the actual experienced and the control R:FR ratio (RFRcontrol is 2.3, related to growth conditions in the validation experiment [31]). Parameter *α* (dimensionless) determines the curvature of the response curve and is referred to as the ‘plasticity strategy’ (See Fig 1 for variation in the curvature related to *α* values). Note that *F* will be 1 when the plasticity strategy *α* is 0, which will not induce petiole or lamina plastic responses. The higher the value of α the more sensitive the genotype is to R:FR decrease. We set a maximum of 2 to *F*, to prevent organs to change their own size more than twice within one day, since this has never been experimentally observed. Variation in the shape of the response curve represents variation in the physiological regulation of the response, as observed among response curves of Arabidopsis mutants [31]. Petiole elongation was simulated by multiplying petiole length with *F*, taking the R:FR perceived by the petiole itself as input [27]. This petiole elongation was calculated every model time-step after simulating petiole growth based on carbon allocation through the relative sink strength principle. Consequently, the petiole increased its demand (sink strength) for carbon substrates for the next time-step to account for the length increase. Lamina growth downregulation was simulated by decreasing the carbon demand (sink strength) (Eq 2):

$$D=\left(\frac{Dp}{F^{n}}\right)$$ (2)

where *D* is the actual lamina carbon demand (mg day^-1^) as affected by R:FR perception, D_p_ the potential lamina carbon demand (mg day^-1^) as calculated by the beta growth function (see above), *F* the relative growth factor based on R:FR perceived by the lamina (calculated with Eq 1), and *n* a dimensionless coefficient that alters the strength of the lamina growth downregulation relative to the petiole elongation response. The default setting of the model had *n* = 2, also referred to as the Average scenario, see **Model scenarios**). An increase of *n* indicates that the demand for lamina growth decreases by which the lamina receives less carbon, while therefore more carbon is available for petiole growth. The *n* value only differed between scenarios. Note that the decreased carbon demand for a given lamina has direct consequences for the absolute carbon allocation to all growing organs; decreased carbon allocation to a lamina can be related to increased carbon allocation to a petiole. Plastic responses could only occur during the growth phase of the specific organ that is set by the beta-growth function.

### Pairwise invasibility plots to determine cESS

In general, evolutionary game theoretical principles assume that over evolutionary time the strategy of a population can change when a rare individual with a different strategy than the population can invade the standing population. A rare mutant with a different strategy than the standing population (also called ‘resident’ population) can invade the standing population when it has a higher performance than the average performance of individuals of the standing resident population, and in theory this invasion will lead to replacement of the population, provided that the change caused to the resident phenotype by the mutant is sufficiently small. With the virtual plant model we captured this by simulating different canopies in which the middle plant of the canopy has the same strategy or a different strategy (also called mutant) than the surrounding resident population (Fig 2). Within this study the different strategies were represented by the shape of the plastic response curve with α ranging from 0 to 0.7 (See Eq 1 and Fig 1). A full matrix of combinations of mutant within a resident population has been simulated, resulting in 64 different canopies (Fig 2C). In total, we replicated these 64 canopies 20 times at five densities and for three scenarios (see below **Model scenarios**), resulting in a total of 19.200 simulations.

The simulated performances of mutants within resident populations were used to calculate the invasion exponents of the mutants, calculated by log(mean(mutant performance)/mean(resident performance)). These invasion exponents resulted in pairwise invasibility plots (Fig 2D). To smooth out stochastic variation simulated by the virtual model, and to be able to get a more detailed estimation of a possible cESS, the simulated performances of the mutants within the resident populations were interpolated with a non-linear smoother after which the invasion components were calculated (i.e. a general additive model [61] using the ‘mgcv’ package in R https://cran.r-project.org/web/packages/mgcv/mgcv.pdf, see S1 Script for details).

### Model scenarios

To explore if selection at different planting densities would result in different cESS, we simulated a full matrix of mutant-resident canopies with plasticity values ranging from 0 to 0.7 at five densities. In addition, we tested how the estimated cESS at different densities alter when changing the strength of lamina responses relative to petiole responses to R:FR. This was done through varying the parameter *n* (in Eq 2), which determined the sink strength of the lamina and therefore the potential lamina growth. In the Average scenario we used the model settings as describe above in which average lamina responses were relatively equal to petiole responses (*n* in Eq 2 is set to 2). In the Weak scenario, we simulated a weak downregulation of the sink strength of the lamina by setting the value of *n* in Eq 2 to 1, and in the Strong scenario we simulated a strong downregulation of the lamina sink strength by setting *n* to 4. Comparing the three scenarios gives insight in the relative importance of the responses that have antagonistic consequences for light capture and therefore plant performance; petiole elongation has generally beneficial consequences by placing the leaf in a higher strata of the canopy versus lamina growth downregulation has generally negative consequences because the size of the lamina that is responsible for capturing light will decrease.

## Supporting information

S1 Fig **Mean leaf angle per plant (A) and canopy leaf area index (B) during canopy development of plants without petiole or lamina plasticity, growing in monomorphic vegetation stands at five planting densities.** In the model, plants increased the angles of individual leaves when the distance with a neighbour leaf was smaller than 2 mm or when the R:FR perception was below 0.5 (see Methods). Leaf area index is based on the total leaf area of the middle plant of the canopy. Data represent mean ± SD (n = 20).
(TIF)

S2 Fig Simulated petiole length and lamina area of individual leaves of plants with different plasticity strategies after growing 46 days in monomorphic vegetation stands at five densities. During canopy development the R:FR ratio within the canopy changed dynamically influencing the growth of the petioles (A-E) and laminas (F-J), depending on their plasticity strategy value (colours, see legend J) and the density (panels in columns). Petiole and lamina sizes are different between densities due to the light availability which drives organ growth. Plants with low plasticity strategy values have relative small petioles and big leaf area, while plants with high plasticity strategies have long petioles and smaller lamina area. Data represent the mean ± SD (n = 20).
(TIF)

S3 Fig Performance values for mutants within resident populations at different planting densities (columns) and different scenarios (rows). The values represent the original calculated total accumulated biomass (in mg) after 46 days of growth (proxy of performance). Different scenarios refer to the balance between petiole and lamina responses. The Average lamina response scenario (A-E) is the default scenario. The Weak (F-J) and Strong (K-O) lamina response scenarios have reduced or increased, respectively, lamina responses compared to petiole responses, although both petiole and lamina plasticity were based on the same plastic response curve. In the model settings these scenarios are created by changing the *n* value in Eq 2 (see Methods); *n* equals 1, 0 or 4 for Average, Weak and Strong scenarios respectively.
(TIF)

S4 Fig Confidence intervals for the calculated invasion exponents for mutants within resident populations at different planting densities (columns) and different scenarios (rows). The values represent the lower and upper range values of the confidence interval, calculated as mean (log(mutant/mean(resident))) ± (sd (log(mutant/mean(resident))) / sqrt(n-1)). Colours correspond to the mean invasion exponent values (presented in Figs 5 and 6), ranging from dark red (negative) to dark blue (positive), while white represents zero. Bold numbers indicate confidence intervals that include zero. Different scenarios refer to the balance between petiole and lamina responses. The Average lamina response scenario (A-E) is the default scenario. The Weak (F-J) and Strong (K-O) lamina response scenarios have reduced or increased, respectively, lamina responses compared to petiole responses, although both petiole and lamina plasticity were based on the same plastic response curve. In the model settings these scenarios are created by changing the *n* value in Eq 2 (see Methods); *n* equals 1, 0 or 4 for Average, Weak and Strong scenarios respectively.
(TIF)

S5 Fig Perceived light by laminas whose petioles perceived a R:FR ratio between 0.95 and 1.05, at five different planting densities. Light is quantified as simulated photosynthetic active radiation (μmol m^-2^ s^-1^). Data used from plants which grew for 46 days in vegetation stands in which all showed no petiole or lamina plasticity (Using the model settings related to the Average scenario). Per planting density a total of 283, 418, 438, 213 and 157 leaves for respectively densities of 100, 400, 711, 1600, 6400 plants m^-2^ were used for this analysis. Triangles illustrate mean, boxplots illustrate median with upper and lower quartile, wiskers at quartile ± 1.5*interquartile range and outliers, created by R v3.2.0.
(TIFF)

S1 Script Interpolation method to create smoothed pairwise invasibility plots. (PDF)
