# Supplementary figures and images for: Variation in plastic responses to light results from selection in different competitive environments—A game theoretical approach using virtual plants
Source: PLoS Comput Biol. 2019 Aug 21;15(8):e1007253. doi: 10.1371/journal.pcbi.1007253 (PMC6703680; doi:10.1371/journal.pcbi.1007253)

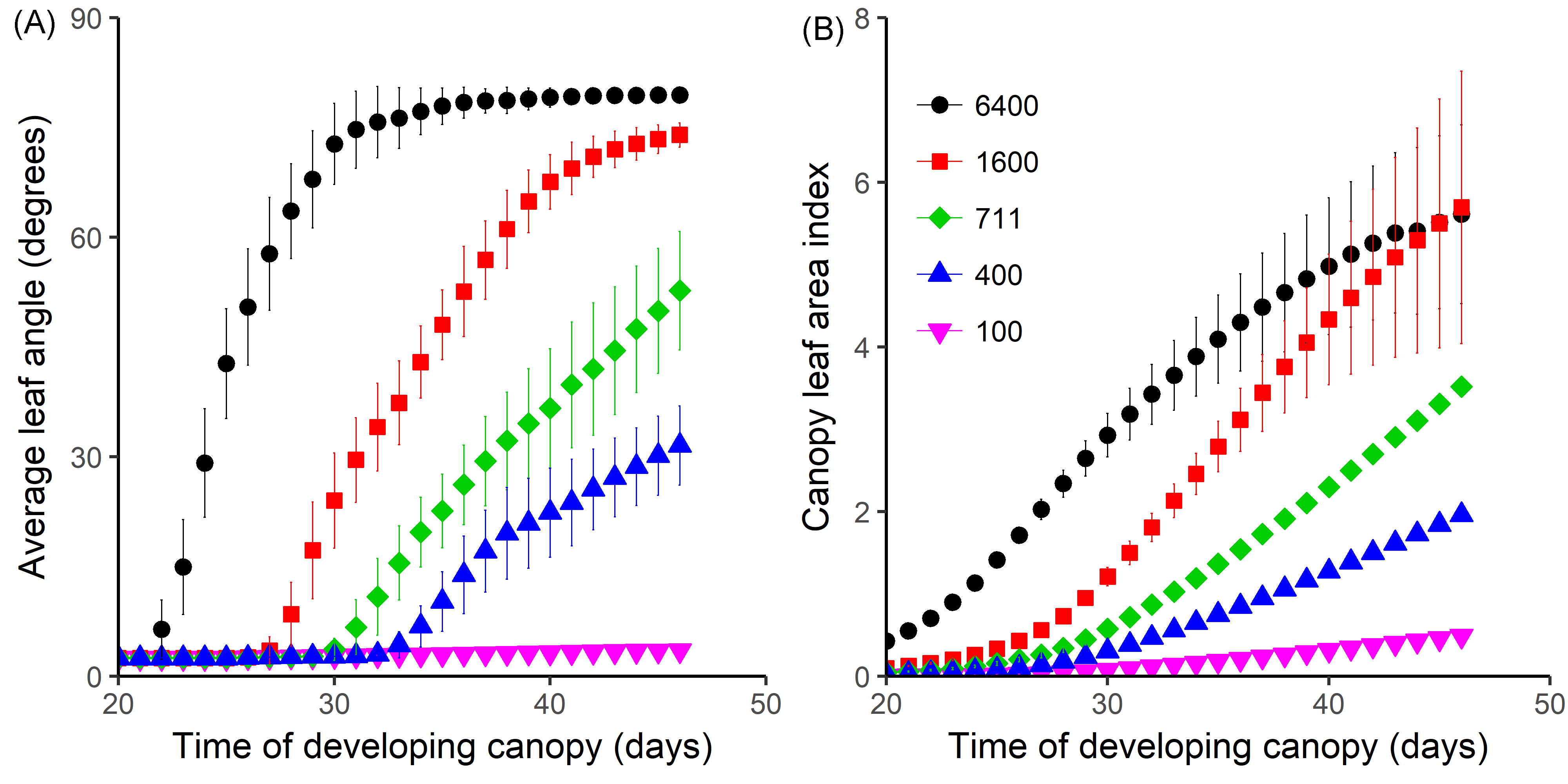

Supplement: S1 Fig — Mean leaf angle per plant (A) and canopy leaf area index (B) during canopy development of plants without petiole or lamina plasticity, growing in monomorphic vegetation stands at five planting densities. In the model, plants increased the angles of individual leaves when the distance with a neighbour leaf was smaller than 2 mm or when the R:FR perception was below 0.5 (see Methods). Leaf area index is based on the total leaf area of the middle plant of the canopy. Data represent mean ± SD (n = 20). (TIF) [file pcbi.1007253.s001.tif]

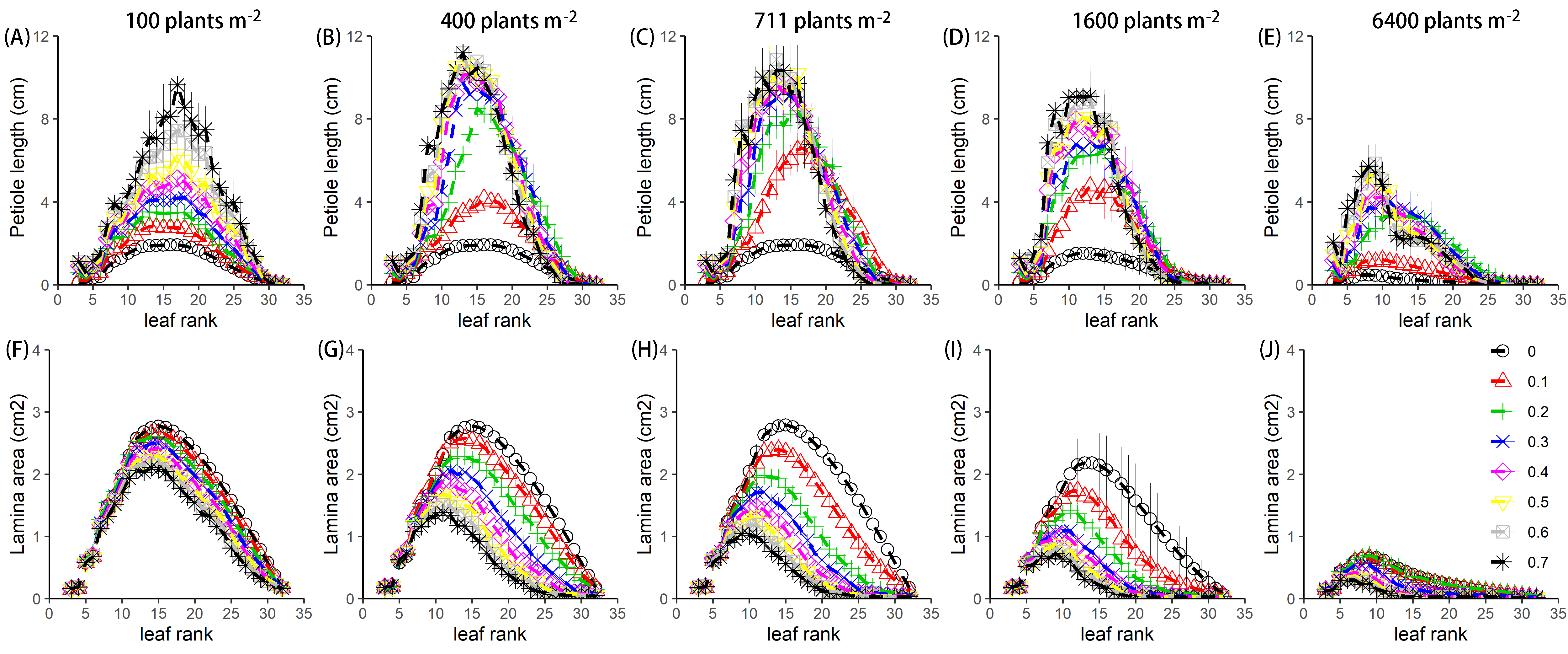

Supplement: S2 Fig — During canopy development the R:FR ratio within the canopy changed dynamically influencing the growth of the petioles (A-E) and laminas (F-J), depending on their plasticity strategy value (colours, see legend J) and the density (panels in columns). Petiole and lamina sizes are different between densities due to the light availability which drives organ growth. Plants with low plasticity strategy values have relative small petioles and big leaf area, while plants with high plasticity strategies have long petioles and smaller lamina area. Data represent the mean ± SD (n = 20). (TIF) [file pcbi.1007253.s002.tif]

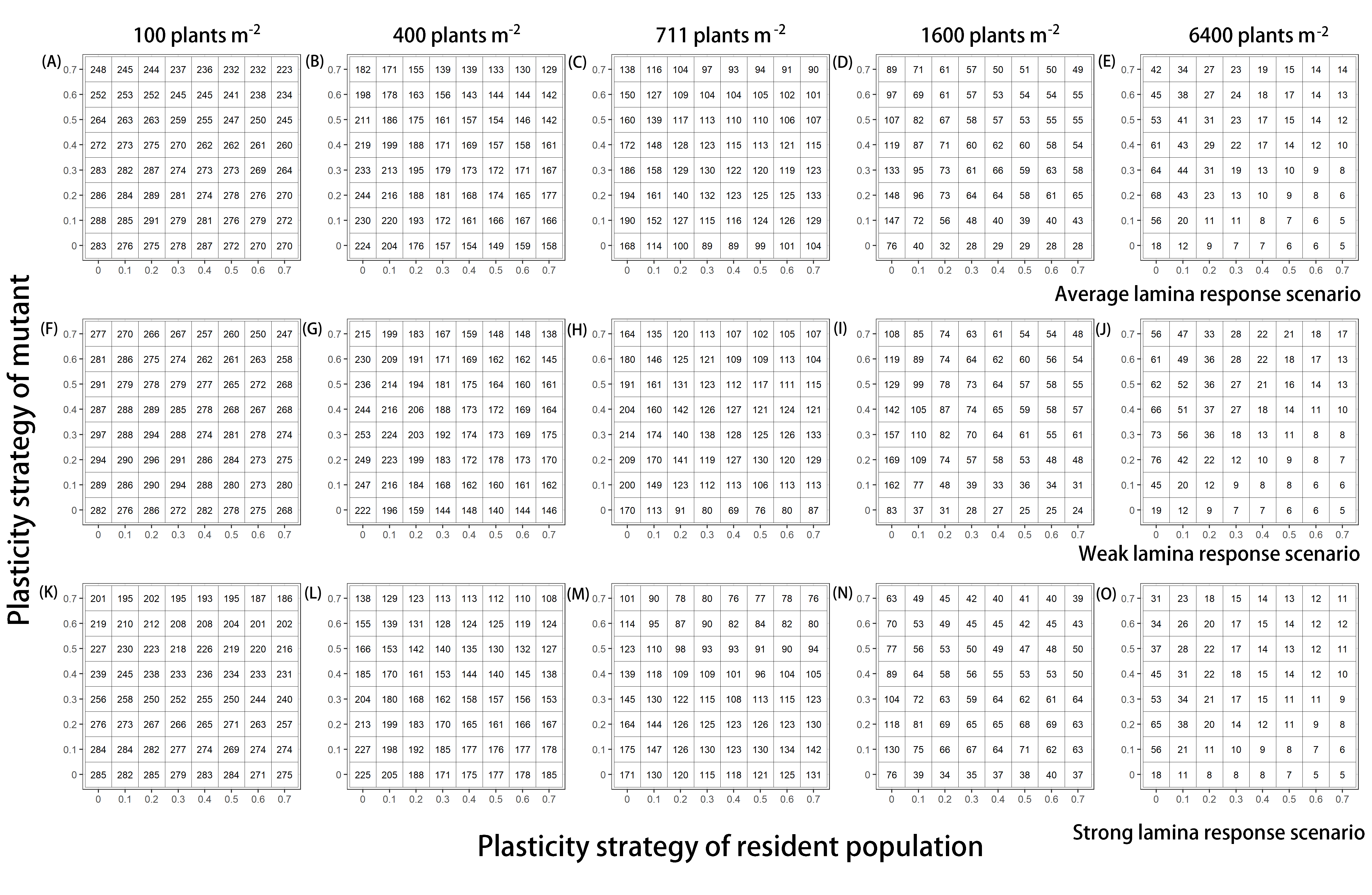

Supplement: S3 Fig — The values represent the original calculated total accumulated biomass (in mg) after 46 days of growth (proxy of performance). Different scenarios refer to the balance between petiole and lamina responses. The Average lamina response scenario (A-E) is the default scenario. The Weak (F-J) and Strong (K-O) lamina response scenarios have reduced or increased, respectively, lamina responses compared to petiole responses, although both petiole and lamina plasticity were based on the same plastic response curve. In the model settings these scenarios are created by changing the n value in Eq 2 (see Methods); n equals 1, 0 or 4 for Average, Weak and Strong scenarios respectively. (TIF) [file pcbi.1007253.s003.tif]

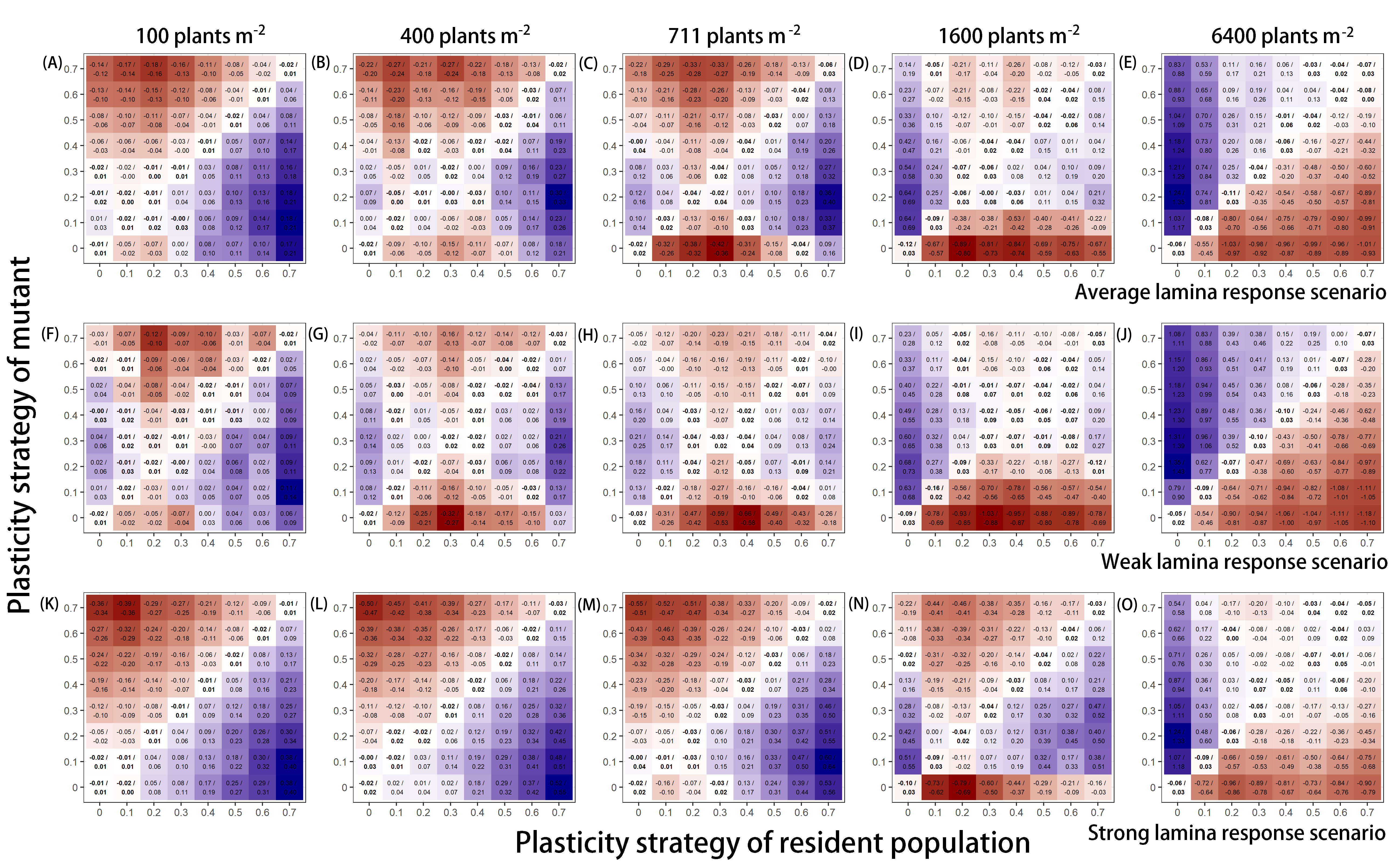

Supplement: S4 Fig — The values represent the lower and upper range values of the confidence interval, calculated as mean (log(mutant/mean(resident))) ± (sd (log(mutant/mean(resident))) / sqrt(n-1)). Colours correspond to the mean invasion exponent values (presented in Figs 5 and 6), ranging from dark red (negative) to dark blue (positive), while white represents zero. Bold numbers indicate confidence intervals that include zero. Different scenarios refer to the balance between petiole and lamina responses. The Average lamina response scenario (A-E) is the default scenario. The Weak (F-J) and Strong (K-O) lamina response scenarios have reduced or increased, respectively, lamina responses compared to petiole responses, although both petiole and lamina plasticity were based on the same plastic response curve. In the model settings these scenarios are created by changing the n value in Eq 2 (see Methods); n equals 1, 0 or 4 for Average, Weak and Strong scenarios respectively. (TIF) [file pcbi.1007253.s004.tif]

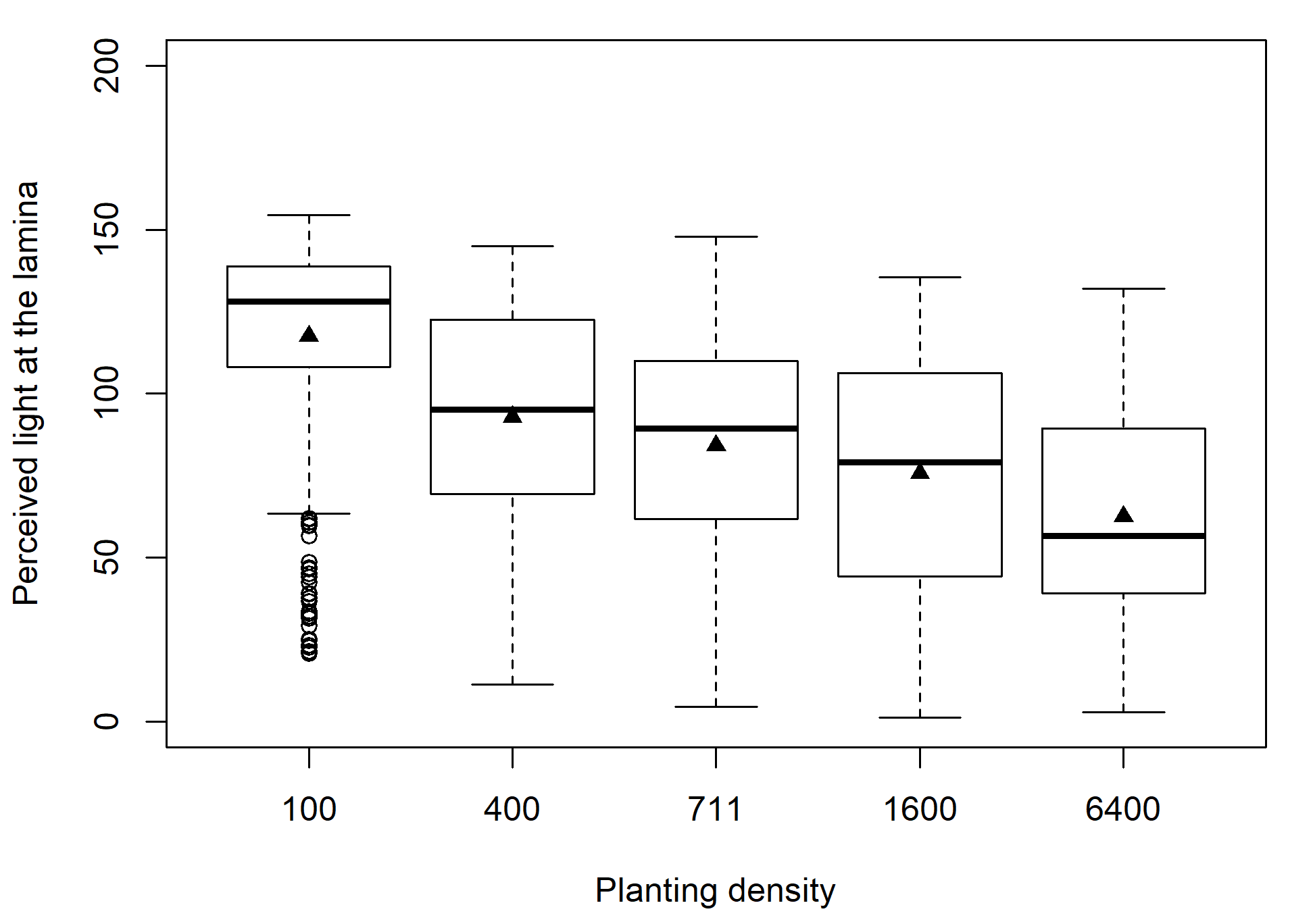

Supplement: S5 Fig — Light is quantified as simulated photosynthetic active radiation (μmol m-2 s-1). Data used from plants which grew for 46 days in vegetation stands in which all showed no petiole or lamina plasticity (Using the model settings related to the Average scenario). Per planting density a total of 283, 418, 438, 213 and 157 leaves for respectively densities of 100, 400, 711, 1600, 6400 plants m-2 were used for this analysis. Triangles illustrate mean, boxplots illustrate median with upper and lower quartile, wiskers at quartile ± 1.5*interquartile range and outliers, created by R v3.2.0. (TIFF) [file pcbi.1007253.s005.tiff]
