# Supplementary material for: Variation in plastic responses to light results from selection in different competitive environments—A game theoretical approach using virtual plants
Source: PLoS Comput Biol. 2019 Aug 21;15(8):e1007253. doi: 10.1371/journal.pcbi.1007253 (PMC6703680; doi:10.1371/journal.pcbi.1007253)

## S1 Script - Interpolation method to create smoothed Pairwise Invasibility Plots

```
library(ggplot2)
library(mgcv) - https://cran.r-project.org/web/packages/mgcv/mgcv.pdf.
library(itsadug)
library(broom)

extract <- function(x){
  d <- c(NA,x$p.table,NA,x$s.table,x$r.sq,x$dev.expl,x$edf,x$n)
#  browser()
  names(d) <-
c(rownames(x$p.table),colnames(x$p.table),rownames(x$s.table),colnames(x$s.table),"R2","Devia
nce explanined","edf","n")
  return(d)
}

extract(summary(gam1))
### take 2 ####
```

**dat = read.csv("Bongers et al. Simulated data.csv")**

```
ipd.un= unique(dat[,c(1,2)])
ipd.un[,2] = rev(ipd.un[,2])
ipd.un[rank(ipd.un$IPD)]

par(mfcol=c(3,5),oma=c(0,0,0,0),mar=c(4,4,4,0.2))

ipd.un[,4:17] <- NA

for (j in 1:nrow(ipd.un)){

  dat.sub = dat[dat$SCEN==ipd.un[j,1] & dat$IPD == ipd.un[j,2],]
  dat.sub$win = NA
  for (i in unique(dat.sub$a1RES)){
    dat.sub[dat.sub$a1RES == i,"win"] = ifelse(dat.sub[dat.sub$a1RES == i,"TBM"]>
dat.sub[dat.sub$a1RES == i & dat.sub$a1FOC==i,"TBM"],1,0)
  }

  gam1 = mgcv::gam(TBM~s(a1RES,a1FOC),data=dat.sub)

  if (j ==1){
    ipd.un[j,4:ncol(ipd.un)] <- extract(summary(gam1))
    colnames(ipd.un)[4:17] <- names(extract(summary(gam1)))
  } else {
    ipd.un[j,4:ncol(ipd.un)] <- extract(summary(gam1))
  }

  summary(gam1)
xval <- seq(-0.05,0.75, length=100)
yval <- seq(-0.05,0.75, length=100)
g1 <- get_predictions(gam1, cond=list(a1RES=xval, a1FOC=yval),
                        rm.ranef=TRUE, print.summary=FALSE)

  g1 <- g1[order(g1$a1RES, g1$a1FOC),]
  zval <- matrix(g1$fit, byrow=TRUE, nrow=100,ncol=100)

  zval.rel = zval
  for (i in 1:100){
    zval.rel[i,] = log(zval[i,]/zval[i,i])
  }
```

```
# color palette
  rP = colorRampPalette(c('dark red','white','dark blue'),bias=1)

  image(xval, yval, zval.rel, col=rP(100),xlab='a1RES',
ylab='a1FOC',main=paste("Density=",round(1/(ipd.un[j,2]^2)*10000),sep=" "),
       breaks=seq(-max(abs(zval.rel)),max(abs(zval.rel)),length.out=101), axes=F)
box()
axis(1, at=seq(0,0.7,0.1), labels=c(0,0.1,0.2,0.3,0.4,0.5,0.6,0.7))
axis(2, at=seq(0,0.7,0.1), labels=c(0,0.1,0.2,0.3,0.4,0.5,0.6,0.7),las=1)

contour(xval, yval, zval.rel , labcex=.8,levels=c(-0.05, -0.1, -0.2, -0.3, -0.4,
                                          -0.6, -0.8, -1.0), col="red",add=T, n=5)
contour(xval, yval, zval.rel , labcex=.8,levels=0, col="black",add=T)
contour(xval, yval, zval.rel , labcex=.8,levels=c(0.05, 0.1, 0.2, 0.3, 0.4,
                                         0.6, 0.8, 1.0), col="blue",add=T, n=5)
}
dev.off()
```
**view("gamoutput”)**

| SCEN | IPD | ess | (Intercept) | Estimate | Std. Error | t value | Pr(>\|t\|) | s(a1RES,a1FOC) | edf | Ref.df | F | p-value | R2 | ance explar | edf | n |
|---|---|---|---|---|---|---|---|---|---|---|---|---|---|---|---|---|
| 1 | 10 | 0.19 | | 264.684 | 0.351 | 753.686 | 1.52E-93 | | 18.210 | 23.219 | 98.551 | 3.43E-264 | 0.973 | 0.981 | 18.210 | 64 |
| 2 | 10 | 0.2 | | 277.400 | 0.511 | 543.112 | 2.61E-97 | | 12.185 | 16.604 | 25.252 | 5.23E-45 | 0.870 | 0.895 | 12.185 | 64 |
| 3 | 10 | 0 | | 242.033 | 0.397 | 610.290 | 1.74E-92 | | 16.463 | 21.476 | 274.128 | 0 | 0.989 | 0.992 | 16.463 | 64 |
| 1 | 5 | 0.235 | | 172.988 | 0.471 | 367.324 | 2.06E-80 | | 17.655 | 22.683 | 135.114 | 0 | 0.980 | 0.986 | 17.655 | 64 |
| 2 | 5 | 0.32 | | 182.553 | 0.409 | 446.569 | 5.40E-78 | | 21.524 | 26.022 | 194.414 | 0 | 0.988 | 0.992 | 21.524 | 64 |
| 3 | 5 | 0 | | 158.231 | 0.383 | 413.148 | 2.71E-80 | | 19.196 | 24.127 | 234.783 | 0 | 0.989 | 0.992 | 19.196 | 64 |
| 1 | 3.75 | 0.225 | | 123.228 | 0.414 | 297.305 | 1.94E-66 | | 24.421 | 27.810 | 121.983 | 0 | 0.982 | 0.989 | 24.421 | 64 |
| 2 | 3.75 | 0.3 | | 129.134 | 0.516 | 250.144 | 1.77E-65 | | 23.029 | 27.037 | 132.139 | 0 | 0.983 | 0.989 | 23.029 | 64 |
| 3 | 3.75 | 0.15 | | 112.707 | 0.383 | 294.378 | 9.20E-67 | | 24.086 | 27.639 | 131.937 | 0 | 0.983 | 0.990 | 24.086 | 64 |
| 1 | 2.5 | 0.3 | | 63.972 | 0.585 | 109.363 | 1.08E-48 | | 25.396 | 28.247 | 64.230 | 4.22E-190 | 0.967 | 0.980 | 25.396 | 64 |
| 2 | 2.5 | 0.45 | | 68.691 | 0.523 | 131.312 | 3.77E-50 | | 26.750 | 28.697 | 134.458 | 0 | 0.984 | 0.991 | 26.750 | 64 |
| 3 | 2.5 | 0.18 | | 58.493 | 0.507 | 115.338 | 3.67E-49 | | 25.769 | 28.389 | 43.376 | 4.05E-123 | 0.951 | 0.971 | 25.769 | 64 |
| 1 | 1.25 | 0.6 | | 21.418 | 0.306 | 69.885 | 2.43E-42 | | 24.301 | 27.750 | 92.373 | 6.46E-279 | 0.976 | 0.985 | 24.301 | 64 |
| 2 | 1.25 | 0.8 | | 24.235 | 0.238 | 101.679 | 1.96E-46 | | 26.455 | 28.615 | 212.950 | 0 | 0.990 | 0.994 | 26.455 | 64 |
| 3 | 1.25 | 0.43 | | 18.009 | 0.256 | 70.276 | 1.46E-40 | | 26.500 | 28.628 | 79.508 | 1.94E-239 | 0.973 | 0.984 | 26.500 | 64 |

```
# test smoothing versus observations
j=1
dat.sub = dat[dat$SCEN==ipd.un[j,1] & dat$IPD == ipd.un[j,2],]
dat.sub$win = NA
for (i in unique(dat.sub$a1RES)){
  dat.sub[dat.sub$a1RES == i,"win"] = ifelse(dat.sub[dat.sub$a1RES ==
i,"TBM"]> dat.sub[dat.sub$a1RES == i & dat.sub$a1FOC==i,"TBM"],1,0)
}

gam1 = mgcv::gam(TBM~s(a1RES,a1FOC),data=dat.sub)
summary(gam1)

g1 <- get_predictions(gam1, cond=list(a1RES=seq(from=0,to=0.7,0.1),
a1FOC=seq(from=0,to=0.7,0.1)),
                 rm.ranef=TRUE, print.summary=FALSE)

g2 = merge(g1,dat.sub[,c("a1RES","a1FOC","TBM")],by=c("a1RES","a1FOC"))

plot(g2$fit~ g2$TBM)
abline(0,1)
```

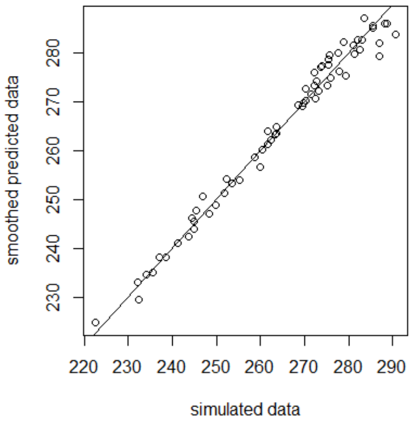

Supplement: S1 Script — (PDF) [file pcbi.1007253.s006.pdf]
